# Phytochemicals of *Conocarpus* spp. as a Natural and Safe Source of Phenolic Compounds and Antioxidants

**DOI:** 10.3390/molecules26041069

**Published:** 2021-02-18

**Authors:** Hanan S. Afifi, Hassan M. Al Marzooqi, Mohammad J. Tabbaa, Ahmed A. Arran

**Affiliations:** 1 Food Research Section, R&D Division, Abu Dhabi Agriculture and Food Safety Authority, Abu Dhabi P.O. Box 52150, United Arab Emirates; hassan.marzouqi@adafsa.gov.ae; 2 Agriculture Research Section, R&D Division, Abu Dhabi Agriculture and Food Safety Authority, Abu Dhabi P.O. Box 52150, United Arab Emirates; Mjtabbaa@ju.edu.jo (M.J.T.); Ahmedarran@hotmail.com (A.A.A.); 3 Department of Animal Production, School of Agriculture, The University of Jordan, Amman 11942, Jordan

**Keywords:** *Conocarpus lancifolius*, *Conocarpus erectus*, response surface methodology, ethanol extracts, polyphenolics, flavonoids, tannins

## Abstract

Optimization of the extraction conditions of polyphenolic compounds for different parts of the Damas species, *Conocarpus lancifolius* and *Conocarpus erectus*, grown under UAE conditions was studied. The combination of ethanol concentration (50, 75, and 100%), temperature (45, 55, and 65 °C) and time (1, 2, and 3 h) was used by applying the Response Surface Methodology. The data showed that the extracts (*n* = 90) contained phenolic compounds, flavonoids, and tannins, and were free of alkaloids. Changing the extraction conditions had a significant effect on the detection of phytosterols, saponins, and glycosides and on the solubility of vanillic acid, *p*-coumaric acid, sinapic acid, *t*-ferulic acid, rutin hydrate, protocatechuic acid, quercetin, and flavone. The data reveal that the roots and leaves of *C. erectus* and the leaves and fruits of *C.* *lancifolius* are the most important plant parts from which to extract these compounds. This study draws attention to the unordinary use of *Conocarpus* spp. as a source of natural food additive.

## 1. Introduction

Maintaining human health is essential. Researchers are therefore continually working to reduce the incidence of the four major non-communicable diseases (NCDs), namely, diabetes, cancer, chronic respiratory diseases, and cardiovascular diseases [1]. Global trends are currently shifting toward consuming healthy, natural, and functional foods and obtaining medicine and bioactive ingredients from natural sources such as plants and microorganisms. For this reason, more attention has been paid to herbal, medicinal, and unordinary plants. Studies have shown that many plants, particularly medicinal plants, contain various secondary metabolites components such as saponins, tannins, alkaloids, phenolics, and flavonoids in their roots, barks, stems, leaves, flowers, and seeds [2,3].

Secondary metabolites are organic molecules, largely generated during the transformation from active to stationary growth, that have an important role in plant defense [4]. The simplest way of classifying these components is into three groups: (1) phenols (such as coumarins, flavonoids, lignins, phenolic acids, stilbens and tannins), (2) terpenes (such as carotenoids, plant volatiles, cardiac glycosides, and sterols), and (3) nitrogen-containing compounds (such as alkaloids and glucosinolates) [5].

Such plant secondary metabolites have been considered for their ability to enhance human health [6]. Some of these components have one of the most influential biological functions, an antioxidant effect, as many are capable of scavenging free radicals directly or indirectly, acting as antioxidants in living cells. Increasing the level of these components during oxidative stress can protect cells from oxidation, in synergy with other antioxidant defense systems [7]. Additionally, polyphenolic compounds are one of the most commonly occurring groups, with a large range of structures, functions, and biological activities [8]. Polyphenolic compounds contribute to color and sensory characteristics [9], as well as the growth and reproduction of plants [10], and provide protection against pathogens and predators [11].

*Conocarpus,* belonging to the Combretaceae family [12], is commonly known as Damas in the Gulf Cooperation Council (GCC) region. It is an evergreen tree distinguished by its resistance to heat and salt and its tolerance to drought. The *C. erectus* and *C. lancifolius*, the two main species of the genus *Conocarpus*, grow in the Gulf countries of the UAE, Yemen, and KSA [13]. The *C. erectus* is native to the mangrove forest ecosystem of Florida in North America, while *C. lancifolius* is native to the coastal and river areas of Somalia and Yemen [14].

Many countries in the world have used *Conocarpus* tree for folk medical applications [15]. It is used as a diuretic and in the treatment of many diseases, such as worms, acute enteritis, colitis, constipation, tooth decay, general infections, malaria, tuberculosis, respiratory diseases, and cancer [16,17], because it has antiplasmodial, antileishmanial, and antitrypanosomal activities [18], as well as antidiabetic potential [19]. In addition, *Conocarpus* shows diverse effects as antibacterial and antifungal toward many pathogenic microorganisms, such as *Staphylococcus aureus*, *Escherichia coli*, *Pseudomonas aeruginosa*, *Bacillus cereus*, *Proteus mirabilis,* and *Klebsella pneumonia* [13,20]. Despite these important applications, the Damas tree is still neglected and has limited usage in Arab countries, where it is only used as a windbreak, for stabilizing sandy soil, and for landscaping. A few years ago, there was a trend to remove these trees due to the serious problems of broken underground water pipes and drainage caused by the long-distance growth and strength of its root network under the soil surface.

Research has shown that the leaves and bark of *Conocarpus* spp. are a source of tannins, which are water-soluble polyphenols that can be used for dyeing purposes. Quercetin-3-*O*-glucoside, kaemferol-3-*O*-glucoside, apigenin, catechin, rutin, quercetin, and quercetin-3-*O*-glucoside-6-*O*-gallic acid have also been identified in the whole plant of *C. erectus* [13]. The amount of phenolic content in *C. erectus* methanol extracts differs according to the plant part. It contains 581.1, 433.9, 236.8, and 216.1 mg/g in its fruits, stems, flowers, and leaves, respectively [21]. The *C. erectus* contains flavonoids and tannins as major phenolic constituents [21]. The methanolic extracts of *C. erectus* stems, leaves, and fruits show a high content of phenolic, flavonoids contents and tannin such as gallic acid, apigenin, catechin, quercetin, quercetin- 3-*O*-glucoside, kaemferol-3-*O*-glucoside, rutin, and quercetin-3-*O*-glucoside-6-*O*-gallic acid [13,21] that possess both bacteriostatic and bactericidal activities [22,23,24].

The methanol extract of *C. lancifolius* has 70.304 ppm chlorogenic acid, 45.772 ppm quercetin, 74.93 ppm ferulic acid, 9 ppm gallic acid, and 57.80 ppm 4-OH 3-methoxy benzoic acid, while the phenolic content of the aerial parts and the roots of *C. lancifolius* methanol extracts reaches 9.78 and 14.01 mg/g, respectively [25]. Additionally, the methanol extract of the stems contains 15.772 ppm *m*-coumaric acid, 14.0149 ppm quercetin, 10.356 ppm chlorogenic acid, 37.108 ppm gallic acid, 9.0325 ppm sinapic acid, and 32.4786 ppm ferulic acid [26].

Little information is known about the polyphenols of the two species of *Conocarpus*, i.e., *C. lancifolius* and *C. erectus*. This is the first study in the GCC area focusing on the effects of the extraction conditions on polyphenols using response surface methodology (RSM) from different parts of trees grown under UAE conditions. A comprehensive research project was conducted to screen the types of active compounds present in ethanolic extracts of roots, leaves, and fruits of *C. lancifolius* and *C. erectus* using different solvent concentrations, temperatures, and extraction times. In addition to quantifying and identifying the types of polyphenols present in the ethanolic extracts, this is also the first time that the extraction conditions of the most important polyphenols extracted from the different parts of the two *Conocarpus* species are optimized.

## 2. Results

### 2.1. Phytochemical Screening of Conocarpus spp.

Solubility is one of the characteristics of components that are often used as a guide for substance applications and to indicate the polarity of the substance [27]. Therefore, three levels of solvent concentrations, temperatures, and extraction times were examined to extract phytochemicals from *C. lancifolius* and *C. erectus* leaves, roots, and fruits. The data show that all extracts of the two species contained phenolic compounds, flavonoids, and tannins. This agrees with the findings of Sundari et al. [28] and Saadullah [29], which refer to the existence of antioxidants in all extracts of various plant parts of Damas trees species (*C. lancifolius* and *C. erectus)*. In contrast, alkaloids disappeared completely from all extracts using all detection methods (Wagner’s and Hager’s). This agrees with the findings of Saadullah et al. [19], who detected the presence of cardiac glycosides, saponins, flavonoids, and tannins and the absence of alkaloids in the methanolic extracts of the aerial parts of *C. lancifolius.* The data show that changing the extraction conditions—i.e., concentration of ethanol, temperature, and extraction time—had a significant effect on the detection of phytosteroids, saponins, and glycosides in plant extracts.

Free phytosteroids can be extracted through solubility in alcohols such as ethanol, as they are insoluble in water and oil. Therefore, the data in Table 1 show that phytosteroids had absolutely vanished from all extracts, except for a few from the *C. lancifolius* fruits and *C. erectus* leaves. These few positive extracts were obtained by using absolute ethanol (100%) in combination with various temperatures (45, 55, or 65 °C) and extraction times (1, 2, or 3 h). Because phytosteroids are associated with plant adaptation to temperature and plant immunity against pathogens, they appear in certain parts of plants (such as leaves and fruits) and are extracted under examined extraction conditions using absolute ethanol only. Saponin contributes to a plant’s protection against microbes and fungi as it has antimicrobial properties, and it is usually found in different parts of the plant such as the leaves, stems, roots, bulbs, blossom, and fruit [30].

The data in Table 1 show that saponins are highly influenced by many factors, as the solubility behavior changes according to plant species, plant part, and extraction conditions. The data show that roots and fruits of *C. lancifolius* were free of saponins, which appeared only in the leaves under different extraction conditions. On the contrary, saponins appeared in all *C. erectus* parts (leaves, fruits and roots), except for a few treatments. Negative results of saponins in *C. erectus* were obtained using ethanol at 50 or 100% and 55 or 65 °C at 1 or 2 h of extraction time.

Regarding glycosides, which are widespread in plants, the qualitative estimation in Table 1 shows that all fruit extracts of both *Conocarpus* species contained glycosides, while different extraction conditions had a different ability to extract glycosides from the roots and the leaves of *C. lancifolius* and *C. erectus*.

### 2.2. Identification of Polyphenols

The extraction procedure of polyphenols, the major class of semi-water-soluble compounds in plants, is significantly influenced by several factors, such as extraction time and type of organic solvent [31,32,33]. Therefore, the interaction of various extraction conditions, including solvent concentration, extraction time, and temperature, was evaluated in order to optimize the extraction conditions of this group of components.

Table 2 shows that 17 components of polyphenols, in addition to tannins, were identified in the extracts. There was a significant difference between *C. lancifolius* and *C. erectus* trees regarding the amount of polyphenols extracted. Moreover, the efficiency of the extraction of polyphenols from *C. erectus* was higher than that of *C. lancifolius,* except for caffeic and chlorogenic acids. Flavone was the highest extracted polyphenols from *C. erectus* and *C. lancifolius* at 278.64 and 123.29 ppm, respectively, and rutin hydrates was the second highest extracted component at 135.31 and 128.56 ppm, respectively. In addition, moderate amounts of quercetin, protocatechuic acid, and sinapic acid were detected in the *C. lancifolius* and *C. erectus* extracts.

A comparison of plant parts (Table 2) shows that the highest extraction of 4-hydroxy benzoic acid (5.63 ppm), vanillic (22.93 ppm), caffeic (8.26 ppm), salicylic (6.23 ppm), 1,2-dihydroxy benzene (4.83 ppm), catechin (4.20 ppm), benzoic acid (4.37 ppm), vanillin (5.63 ppm), and cinnamic acid (4.22 ppm) was obtained from the roots. However, *p*-coumaric (20.01 ppm), *t*-ferulic (29.67 ppm), sinapic acid (57.03 ppm), chlorogenic acid (6.59 ppm), vanillin (5.68 ppm), rutin hydrate (190.21 ppm), and flavones (397.37 ppm) reached the highest level in leaf extracts. The fruit extracts contained the maximum content of protocatechuic acid (103.31 ppm) and quercetin (106.82 ppm). Otherwise, there were no differences between plant parts regarding the amount of extracted tannins.

On the contrary, a comparison of the amount of polyphenols among the fruit, leaf and root extracts of the two species of *Conocarpus* (Table 3) shows that the root extracts of *C. erectus* were the most abundant, containing a high level of nine polyphenols: 4-hydroxy benzoic acid, vanillic, salicylic, 1,2-dihydroxy benzene, catechin, benzoic acid, vanillin, *p*-cinnamic acid and quercetin. This was followed by the leaf extracts of the same species, containing the highest level of four components: *p*-coumaric, *t*-ferulic acid, rutin hydrate, and flavones. Meanwhile, fruits contained the highest level of quercetin and tannins. In contrast, the extracts of *C. lancifolius* leaves and fruits contained the highest level of only two components, namely, sinapic and chlorogenic acids and caffeic and protocatechuic acids, respectively. Accordingly, the data show that the most extracted phenolic compounds were vanillic acid, *p*-coumaric acid, sinapic acid, rutin hydrate, *t*-ferulic acid, protocatechuic acid, quercetin, and flavone. Consequently, the focus is on these eight compounds in terms of the impact of the extraction condition.

According to the results in Table S1, Supplementary Materials, vanillic acid, which is widely used as a food additive and flavoring and in perfumery, was one of the most response variables extracted from different parts of *Conocarpus* species. Moreover, the data indicate that the roots of both *C. lancifolius* and *C. erectus* were the best plant part from which to extract vanillic acid, using 50% ethanol at 45 °C for 2 h to obtain 44.48 and 124.79 ppm, respectively. The data in Table S2 indicate that the highest concentration of *p*-coumaric acid, which plays an important role in human health due to its antimicrobial and antioxidant properties, was extracted from *C. erectus* roots (235.06 ppm) using 50% ethanol at 45 °C for 2 h and its leaves (134.14 ppm) using 100% ethanol, at 55 °C for 1 h. *t*-Ferulic acid, a potent free-radical scavenger and antioxidant, was extracted mostly from *C. erectus* leaves with an average of 175.20 ppm using an absolute ethanol at 55 °C for 3 h, followed by extraction from *C. lancifolius* roots (137.07 ppm) using 50% ethanol at 45 °C for 2 h, as shown in Table S3. The data in Table S4 show the extraction level of one of the important antioxidant, antimicrobial, anti-inflammatory, and anticancer component, sinapic acid. The data revealed that 254.54 ppm of sinapic acid was extracted using 100% ethanol at 45 °C for 2 h from *C. erectus* leaves, while 237.54 ppm was extracted from *C. lancifolius* roots using 50% ethanol at 45 °C for 2 h. The data in Table S5 indicate that rutin hydrate was highly extracted (1362.55 ppm) from *C. erectus* leaves using absolute ethanol at 45 °C for 3 h, while 183.40 ppm of protocatechuic acid and 137.31 ppm of quercetin were extracted from *C. lancifolius* fruits and *C. erectus* roots, respectively, using 50% ethanol at 45 °C for 2 h (Tables S6 and S7). The data in Table S8 show that flavone was the highest detected polyphenol in all ethanolic extracts of the *Conocarpus* species and its parts. This component is vital for both plant and human, as it acts as a UVB protectant and natural pesticide in plants and plays a role in antioxidant, antimicrobial, anti-tumor, antiproliferative, and anti-inflammatory activities for humans [34,35,36].

The results show that the Damas tree has added value. It can be used as a source of active ingredients that can be used in many applications, such as in functional foods and food supplements. Each species can be used for the extraction of specific components. *C. erectus* can be used as a source of vanillic, *p*-coumaric, quercetin, rutin hydrate, and flavone, while *C. lancifolius* can be used as a source of *t*-ferulic acid, sinapic acid, and protocatechuic acid.

**Figure 1:**
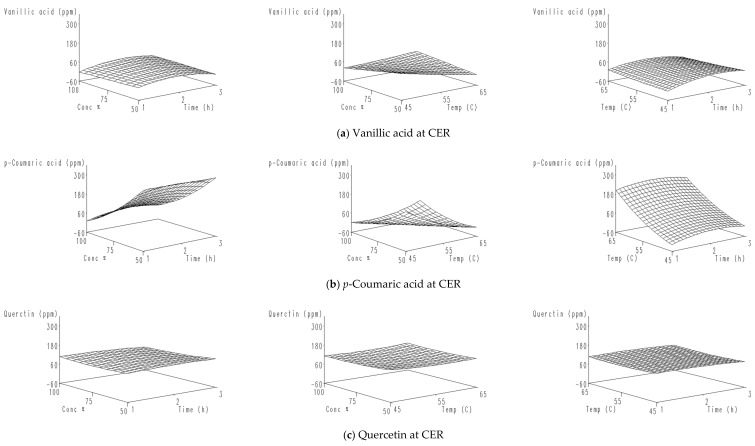

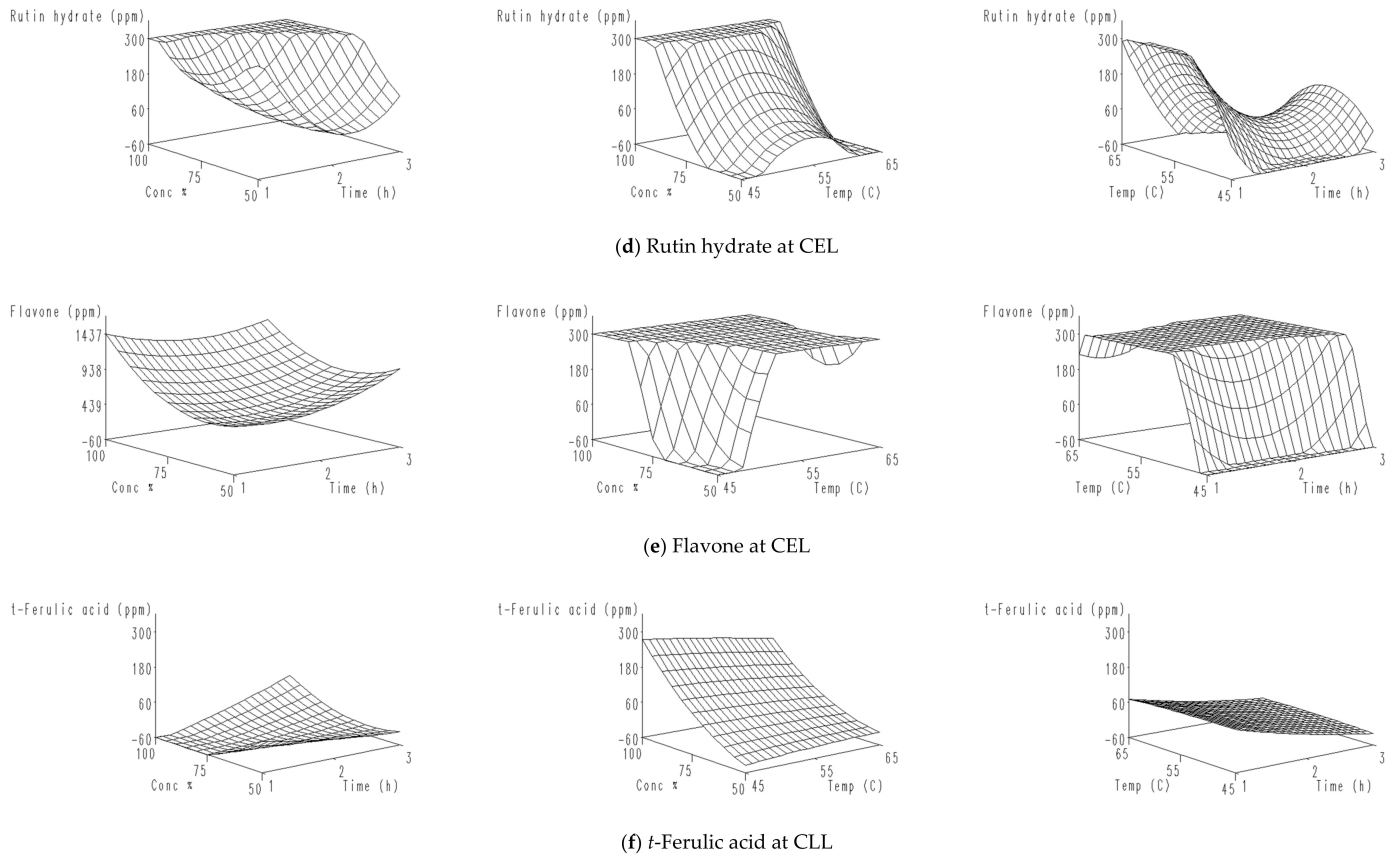

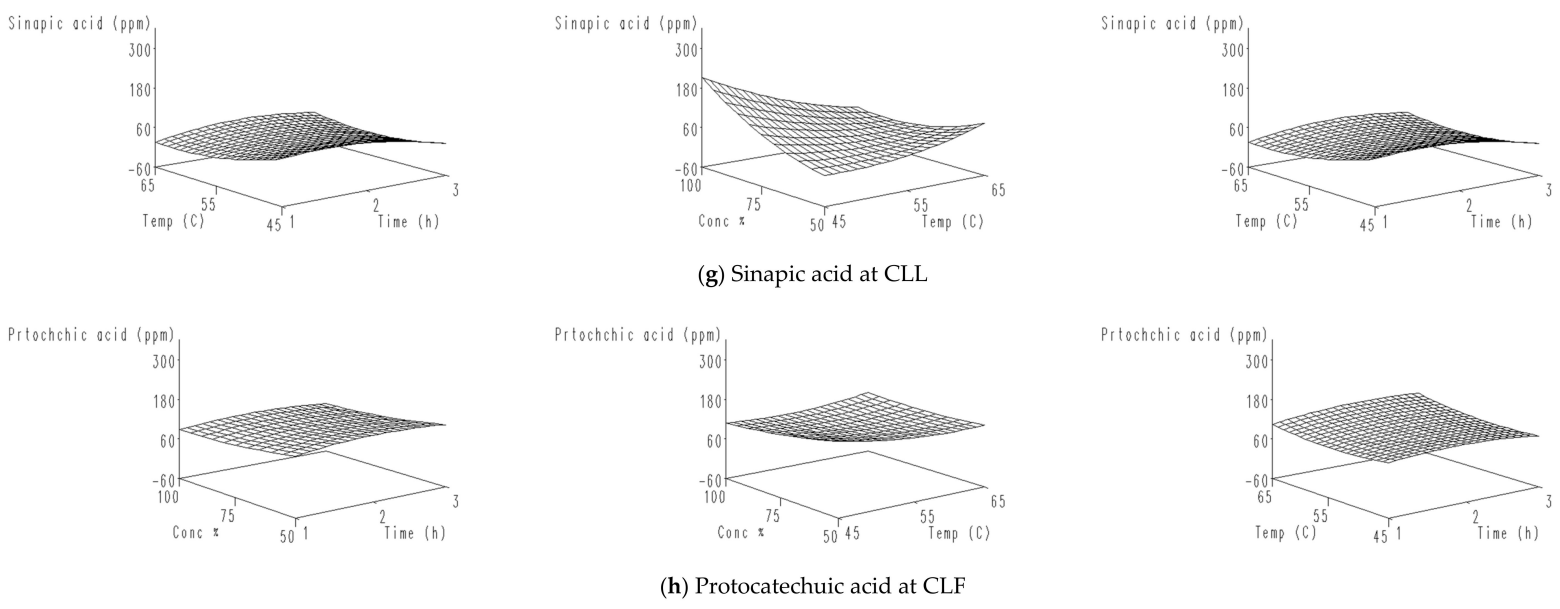
Response surface plots illustrating the effects of different extraction conditions on the yield of (**a**) Vanillic acid from *C. erectus* roots, (**b**) *p*-Courmaric acid from *C. erectus* roots, (**c**) Quercetin from *C. erectus* roots, (**d**) Rutin hydrate from *C. erectus* leaves, (**e**) Flavone from *C. erectus* leaves; (**f**) *t*-Ferulic acid from C. *lancifolius* leaves; (**g**) Sinapic acid from *C. lancifolius* leaves; and (**h**) Protocatechuic acid from *C. lancifolius* fruits.

A three-dimensional drawing of the response surfaces that illustrate the relationship between the independent and dependent (polyphenols) variables is shown in Figure 1.

The data in Figure 1a show that the rise in both ethanol concentration and extraction time and temperature resulted in a significant increase in vanillic acid. The increase in ethanol concentration and temperature, on the contrary, contributed to a strong decrease in vanillic acid.

Figure 1b shows that the *p*-coumaric acid yield increased with low ethanol concentration (50%) and low temperature (45 °C), high temperature (65 °C) and high extraction time (3 h), or with long extraction time (3 h), and low ethanol concentration (50%). The relationship between the concentration of ethanol and the extraction time, and between the temperature and the extraction time, increased the amount of quercetin extracted (Figure 1c), although decreasing both the concentration of ethanol and the temperature caused an increase in the extraction of quercetin. The level of rutin hydrate varied with the interaction between the concentration of ethanol and the extraction time. Figure 1d shows that an increase in the ethanol concentration induced a sharp increase in the amount of extracted rutin hydrate. Moreover, an increase in temperature and extraction time also increased the extraction of rutin hydrate. The polyphenol with the highest amount extracted from *Conocarpus* spp. was flavone. Figure 1e indicates that flavone increased significantly with an increase in ethanol concentration above 75% and a temperature above 55 °C, although increasing the temperature from 55 to 65 °C sharply increased the extraction of flavone with changes in the extraction time. This is due to an increase in solvent penetration at high temperatures and the greater solubility with an increase in solvent concentration [37]. On the contrary, an increase in the ethanol concentration above 75% with an increase in the extraction time, as well as increase in the temperature with a decrease in the extraction time, caused an increase in the amount of extracted *t*-ferulic acid (Figure 1f), while decreasing the concentration of ethanol strongly reduced the extraction of *t*-ferulic acid with changing temperature. Figure 1g shows that increasing the concentration of ethanol and the time or temperature of extraction produced an increase in the yield of sinapic acid, while the yield increased when using a low temperature (45 °C) and a short extraction time (1 h). Increasing the extraction time and concentration of ethanol positively influenced the extracted amount of protocatechuic acid. In addition, the interaction of low temperature with low time caused an increase in the yield of protocatechuic acid, while the interaction between different levels of temperature and the extraction time caused slight differences in the yield of protocatechuic acid (Figure 1h).

Table 4 shows the influence of the three independent variables on the optimization of the most important polyphenols (vanillic acid, *p*-coumaric acid, quercetin, rutin hydrate, flavone, *t*-ferulic acid, Sinapic acid, and protocatechuic acid) of the *Conocarpus* species in order to effectively isolate and utilize these compounds of interest. No previous studies on the optimization of the conditions of extraction of active components from different parts of Damas tree (*C. lancifolius* and *C. erectus)* have been published for comparison.

The data show that the roots and leaves of the *C. erectus* and the leaves and fruits of the *C. lancifolius* are the most important parts of the plant. The minimum solvent concentration (ethanol) that can be used for extraction is 48%, while most of the active components can be extracted by using an ethanol concentration greater than 70%, at around 57 °C for approximately 2 h.

## 3. Materials and Methods

### 3.1. Chemicals

Absolute ethanol was acquired from Panreac (Barcelona, Spain) for extraction. HPLC-grade solvent, standards of respective polyphenols, and other chemicals of high purity were obtained from Sigma-Aldrich-Fluka Co. Ltd. (St. Louis, MO, USA), unless otherwise specified. High-purity water was prepared using a Milli Q purification system (Peenya, Bangalore, India).

### 3.2. Collecting of Plant Materials and Extraction Procedure

Plant taxonomy and tree measurements of the *C. lancifolius* and *C. erectus* were identified according to Wood [38] (Table S9). Ten kilograms of fully matured fruits, leaves, and roots were collected locally from Al Salamat Research Station, Al Ain, UAE in March 2016. The plant materials were washed, plant parts deployed, and the individual parts were dried completely under shade conditions for 12–15 days on a clean, dry surface, then ground using an electric mill (Retsch, Germany) to a 300–500 µm fine powder. The powder was packed in plastic bags and stored in a dark, cold place until the extraction process.

The maceration method was applied using different extraction conditions, as shown in Figure S1, for two extraction cycles and filtration steps to optimize the efficiency of the extraction of the potential bioactive components. The solvent was removed from extracts using a rotary vacuum evaporator (Bibby Sterilin, model RE-200, Staffordshire, UK) at 40 °C. The crude extracts of all plant parts were kept in dark glass bottles inside the fridge at 5 °C until analysis.

### 3.3. Experimental Design

Optimization of the extraction conditions of the polyphenols from powder of different parts of *C. lancifolius* and *C. erectus* was carried out using a central composite design (CCD) and response surface methodology (RSM) according to Myers and Montgomery [39,40]. Three independent variables, X_1_—solvent concentration, X_2_—extraction temperature, and X_3_—extraction time, were studied, as shown in Table S10. For the three factors, a three-level face-centered cube design was applied, consisting of 15 experimental runs with three replicates at the central point [41,42]. All experiments were conducted in random order to eliminate error due to the extraneous factors.

### 3.4. Phytochemicals Screening Tests

Phytochemical compounds, including phenolic compounds, flavonoids, tannins, alkaloids, phytosteroids, saponins, and glycosides, were detected in a total of 90 *C. lancifolius* and *C. erectus* ethanolic extracts, as mentioned by Brain and Turner [43] and Evans et al. [44].

### 3.5. Determination of Polyphenolic Components Using HPLC

The secondary metabolite polyphenols were analyzed in 15 extracts of *C. lancifolius* and *C. erectus* belonging to the fruits, leaves, and roots individually using HPLC. A column of Hypersil ODS (100 × 4.6 mm, 3 µm) with a 30 µL injection volume was used. The three mobile phases used were as follows: (A) 30 mM ammonium acetate pH 4.7, (B) methanol, and (C) acetonitrile in different ratios (61:19.5:19.5, 32:34:34, and 5:47.5:47.5 *v*/*v*/*v*), at a flow rate of 1.1 mL/min with UV detection at 210 nm wavelength. The following program was applied: 100% A for 5 min, followed by a linear gradient to 100% B in 5 min, followed by a linear gradient to 100% C for 5 min, and then isocratic for 5 min before equilibrium. The accurate weight of each component was diluted with a diluting solvent, i.e., acetonitrile, in a volumetric flask for the preparation of standard stock solutions. Serial dilutions ranging from 10 to 200 µg/mL with a mobile phase at the appropriate concentration were prepared. To ensure reliable results, validation of the method, including precision, accuracy, linearity, limit of detection (LOD), limit of quantification (LOQ), and specificity, was conducted. A summary of the HPLC method validation is shown in Tables S11 and S12. All solutions were remaining stable at 4 °C for at least 15 days, and their stability was checked at regular intervals using HPLC. The daily preparation of fresh solutions was carried out by diluting the stock solution with the diluting solvent (mobile phase A). The retention time of the detected components is illustrated in Table S13 and Figure S2.

### 3.6. Determination of Tannins

The content of tannins was measured in triplicate in ethanolic extracts of the two species of *Conocarpus*, using the method mentioned by Fagbemi et al. [45].

### 3.7. Statistical Analysis

Response surface methodology (RSM) was used to build and evaluate the obtained results using Minitab Software (Minitab version 16.0, Minitab Inc., State College, PA, USA) for three independent variables. In addition, SAS version 2004 was used to compare treatment means in two steps: (1) the GLM procedure was used to analyze variance for balanced data of plant species and extortion conditions, and (2) the RSREG procedure was used for the response surface regression analysis. Means, presented as means ± standard deviations, and significant effects were compared using two-tailed t-tests at *p* < 0.05.

## 4. Conclusions

In conclusion, the results indicate that the tested extraction conditions were effective for isolating some important polyphenols from *Conocarpus* species including vanillic acid, *p*-coumaric acid, quercetin, rutin hydrate, flavone, *t*-ferulic acid, sinapic acid, and protocatechuic acid. The roots and leaves of *C. erectus* and the leaves and fruits of *C. lancifolius* were the most important sources of these components. These are promising results for the use of these trees as a source of bioactive components that can be used for pharmaceutical purposes and for the production of functional foods. More investigation is still needed regarding the optimization extraction conditions.

## Figures and Tables

**Table 1:** Phytochemical screening of *Conocarpus lancifolius* and *Conocarpus erectus* extracts from leaves, fruits, and roots using RSM.

Standard Order.	Coded and Actual Level of Variables			Leaves						Fruits						Roots					
				*C. lancifolius*			*C. erectus*			*C. lancifolius*			*C. erectus*			*C. lancifolius*			*C. erectus*		
	Solvent Conc.	Temp.	Time	Phytosteroids	Saponins	Glycosides	Phytosteroids	Saponins	Glycosides	Phytosteroids	Saponins	Glycosides	Phytosteroids	Saponins	Glycosides	Phytosteroids	Saponins	Glycosides	Phytosteroids	Saponins	Glycosides
1	1 (100)	1 (65)	0 (2)	-ve	+	-ve	-ve	+	-ve	+	-ve	+	-ve	-ve	+	-ve	-ve	+	-ve	+	+
2	−1 (50)	0 (55)	1 (3)	-ve	+	-ve	-ve	+	-ve	-ve	-ve	+	-ve	-ve	+	-ve	-ve	-ve	-ve	+	+
3	0 (75)	0 (55)	0 (2)	-ve	+	+	-ve	+	-ve	-ve	-ve	+	-ve	-ve	+	-ve	-ve	+	-ve	+	-ve
4	0 (75)	1 (65)	1 (3)	-ve	+	-ve	-ve	+	-ve	-ve	-ve	+	-ve	-ve	+	-ve	-ve	+	-ve	+	-ve
5	1 (100)	0 (55)	−1 (1)	-ve	+	+	+	-ve	-ve	+	-ve	+	-ve	+	+	-ve	-ve	+	-ve	+	-ve
6	1 (100)	−1 (45)	0 (2)	-ve	-ve	+	+	+	-ve	+	-ve	+	-ve	+	+	-ve	-ve	+	-ve	-ve	-ve
7	−1 (50)	1 (65)	0 (2)	-ve	+	-ve	-ve	-ve	-ve	-ve	-ve	+	-ve	-ve	+	-ve	-ve	+	-ve	+	-ve
8	0 (75)	0 (55)	0 (2)	-ve	+	+	-ve	+	-ve	-ve	-ve	+	-ve	-ve	+	-ve	-ve	+	-ve	+	-ve
9	0 (75)	1 (65)	−1 (1)	-ve	+	-ve	-ve	+	-ve	-ve	-ve	+	-ve	-ve	+	-ve	-ve	+	-ve	-ve	+
10	1 (100)	0 (55)	1 (3)	-ve	+	+	+	+	-ve	+	-ve	+	-ve	-ve	+	-ve	-ve	+	-ve	-ve	+
11	0 (75)	−1 (45)	1 (3)	-ve	+	+	-ve	+	-ve	-ve	-ve	+	-ve	-ve	+	-ve	-ve	+	-ve	+	+
12	0 (75)	0 (55)	0 (2)	-ve	+	+	-ve	+	-ve	-ve	-ve	+	-ve	-ve	+	-ve	-ve	+	-ve	+	-ve
13	−1 (50)	0 (55)	−1 (1)	-ve	+	-ve	-ve	+	-ve	-ve	-ve	+	-ve	-ve	+	-ve	-ve	+	-ve	+	+
14	0 (75)	−1 (45)	−1 (1)	-ve	+	-ve	-ve	+	-ve	-ve	-ve	+	-ve	-ve	+	-ve	-ve	+	-ve	+	+
15	−1 (50)	−1 (45)	0 (2)	-ve	+	-ve	-ve	+	-ve	-ve	-ve	+	-ve	-ve	+	-ve	-ve	+	-ve	+	+

+—presence; -ve—absence. Levels of independent variables (Solvent concentration, %, Temperature, °C and Time, h) are shown in Table S10, Supplementary Materials.

**Table 2:** Content of polyphenols (ppm) of *Conocarpus* species and its parts (leaves, fruits and roots) extracts.

Plant	4-Hydroxy Benzoic Acid	Vanillic Acid	Caffeic Acid	Salicylic Acid	1,2-Dihydroxy Benzene	Catechin	Benzoic Acid	*p*-Coumaric Acid	*t*-Ferulic Acid
**CE**	3.33 ^a^	12.54 ^a^	4.60 ^b^	3.26 ^a^	2.33 ^a^	1.89 ^a^	2.21 ^a^	16.21 ^a^	15.12 ^a^
**CL**	1.87 ^b^	7.32 ^b^	6.04 ^a^	0.99 ^b^	1.02 ^b^	1.40 ^b^	1.32 ^b^	11.48 ^b^	10.73 ^b^
**LSD**	**0.14**	**0.28**	**0.23**	**0.03**	**0.03**	**0.07**	**0.07**	**0.52**	**0.44**
**Plant**	**Sinapic** **Acid**	**Chlorogenic** **Acid**	**Vanillin**	**Rutin** **Hydrate**	**Cinnamic** **Acid**	**Protocatechuic** **Acid**	**Quercetin**	**Flavone**	**Tannins**
**CE**	32.62 ^a^	2.33 ^b^	4.91 ^a^	135.31 ^a^	2.13 ^a^	57.25 ^a^	79.31 ^a^	278.64 ^a^	0.56 ^a^
**CL**	28.03 ^b^	5.89 ^a^	3.18 ^b^	128.56 ^b^	1.24 ^b^	56.24 ^a^	67.41 ^b^	123.29 ^b^	0.43 ^b^
**LSD**	**1.34**	**0.22**	**0.17**	**4.67**	**0.10**	**2.31**	**0.96**	**4.88**	**0.09**
**Plant Part**	**4-Hydroxy** **Benzoic Acid**	**Vanillic** **Acid**	**Caffeic** **Acid**	**Salicylic** **Acid**	**1,2-Dihydroxy** **Benzene**	**Catechin**	**Benzoic** **Acid**	***p*-Coumaric** **Acid**	***t*-Ferulic** **Acid**
**L**	1.80 ^b^	2.42 ^c^	1.18 ^c^	0.15 ^b^	0.17 ^b^	0.62 ^b^	0.67 ^b^	20.01 ^a^	29.67 ^a^
**F**	0.37 ^c^	4.43 ^b^	6.60 ^b^	0.00 ^c^	0.03 ^c^	0.12 ^c^	0.25 ^c^	8.43 ^c^	0.94 ^c^
**R**	5.63 ^a^	22.93 ^a^	8.26 ^a^	6.23 ^a^	4.83 ^a^	4.20 ^a^	4.37 ^a^	13.09 ^b^	8.17 ^b^
**LSD**	**0.17**	**0.34**	**0.27**	**0.03**	**0.03**	**0.08**	**0.08**	**0.63**	**0.54**
**Plant Part**	**Sinapic** **Acid**	**Chlorogenic** **Acid**	**Vanillin**	**Rutin** **Hydrate**	**Cinnamic** **Acid**	**Protocatechuic** **Acid**	**Quercetin**	**Flavone**	**Tannins**
**L**	57.03 ^a^	6.59 ^a^	5.68 ^a^	190.21 ^a^	0.41 ^b^	30.89 ^c^	16.75 ^c^	397.37 ^a^	0.50 ^a^
**F**	13.68 ^c^	0.31 ^c^	0.83 ^b^	89.48 ^c^	0.43 ^b^	103.31 ^a^	106.82 ^a^	96.78 ^c^	0.51 ^a^
**R**	20.27 ^b^	5.44 ^b^	5.63 ^a^	116.11 ^b^	4.22 ^a^	36.04 ^b^	96.52 ^b^	108.73 ^b^	0.48 ^a^
**LSD**	**1.65**	**0.26**	**0.21**	**5.72**	**0.12**	**2.83**	**1.17**	**5.97**	**0.11**

Whereas CL is *C. lancifolius,* CE is *C. erectus* and L = leaves, F = fruits, R = roots. LSD is the Least Significant Difference. Means with different letters within the same column are significantly different *p* ≤ 0.05.

**Table 3:** Polyphenols of fruits, leaves, and roots of *C. erectus* and *C. lancifolius* extracts.

Part	4-Hydroxy Benzoic Acid	Vanillic Acid	Caffeic Acid	Salicylic Acid	1,2-Dihydroxy Benzene	Catechin	Benzoic Acid	*p*-Coumaric Acid	*t*-Ferulic Acid
**CEL**	2.49 ± 2.75 ^c^	3.47 ± 3.34 ^d^	1.69 ± 2.21 ^d^	0.30 ± 1.16 ^c^	0.18 ± 0.20 ^c^	0.30 ± 0.29 ^d^	0.75 ± 0.47 ^c^	23.72 ± 41.41 ^a^	32.74 ± 27.58 ^a^
**CER**	6.83 ± 20.2 1^a^	27.45 ± 33.66 ^a^	11.29 ± 26.63 ^b^	9.49 ± 35.91 ^a^	6.76 ± 0.15 ^a^	5.30 ± 18.87 ^a^	5.66 ± 20.50 ^a^	18.55 ± 58.64 ^b^	11.07 ± 34.18 ^c^
**CEF**	0.67 ± 1.40 ^e^	6.70 ± 5.81 ^c^	0.98 ± 1.20 ^e^	0.002 ± 0.01 ^d^	0.05 ± 0.37 ^d^	0.09 ± 0.23 ^e^	0.22 ± 0.30 ^e^	6.37 ± 13.62 ^f^	1.57 ± 1.89 ^e^
**CLL**	1.12 ± 2.04 ^d^	1.38 ± 2.22 ^f^	0.68 ± 1.76 ^e^	0.00 ± 0.00 ^d^	0.16 ± 0.37 ^c^	0.94 ± 0.92 ^c^	0.59 ± 0.63 ^d^	16.30 ± 21.61 ^c^	26.59 ± 51.00 ^b^
**CLR**	4.43 ± 11.61 ^b^	18.42 ± 11.57 ^b^	5.23 ± 10.19 ^c^	2.97 ± 11.25 ^b^	2.90 ± 10.18 ^b^	3.10 ± 10.63 ^b^	3.09 ± 11.19 ^b^	7.64 ± 11.70 ^e^	5.27 ± 12.37 ^d^
**CLF**	0.07 ± 0.37 ^f^	2.15 ± 6.36 ^e^	12.22 ± 10.29 ^a^	0.00 ± 0.00 ^d^	0.01 ± 0.04 ^d^	0.15 ± 0.17 ^e^	0.27 ± 0.23 ^c^	10.49 ± 12.46 ^d^	0.32 ± 0.63 ^f^
**Part**	**Sinapic** **Acid**	**Chlorogenic** **Acid**	**Vanillin**	**Rutin** **Hydrate**	**Cinnamic** **Acid**	**Protocatechuic** **Acid**	**Quercetin**	**Flavone**	**Tannins**
**CEL**	55.73 ± 47.29 ^b^	0.44 ± 1.23 ^d^	4.97 ± 4.27 ^c^	214.32 ± 328.17 ^a^	0.72 ± 0.64 ^c^	33.28 ± 36.65 ^d^	19.70 ± 35.30 ^d^	593.14 ± 642.06 ^a^	0.53 ± 0.10 ^b^
**CER**	25.40 ± 59.72 ^c^	5.93 ± 18.01 ^b^	8.31 ± 14.52 ^a^	178.21 ± 240.39 ^b^	5.25 ± 18.85 ^a^	43.53 ± 26.00 ^c^	109.68 ± 10.66 ^a^	135.19 ± 56.44 ^c^	0.55 ± 0.18 ^b^
**CEF**	16.73 ± 19.01 ^d^	0.62 ± 2.23 ^d^	1.46 ± 2.05 ^e^	13.39 ± 10.14 ^e^	0.44 ± 0.10 ^d^	94.93 ± 33.87 ^b^	108.57 ± 28.80 ^a^	107.58 ± 21.06 ^d^	0.60 ± 0.13 ^a^
**CLL**	58.33 ± 58.72 ^a^	12.74 ± 20.26 ^a^	6.39 ± 3.76 ^b^	166.10 ± 156.02 ^c^	0.10 ± 0.30 ^e^	28.49 ± 26.12 ^e^	13.80 ± 26.13 ^e^	201.60 ± 215.75 ^b^	0.47 ± 0.27 ^c^
**CLR**	15.14 ± 20.94 ^d^	4.94 ± 9.15 ^c^	2.95 ± 9.97 ^d^	54.00 ± 56.54 ^d^	3.20 ± 10.83 ^b^	28.54 ± 24.96 ^e^	83.36 ± 48.76 ^c^	82.27 ± 25.57 ^e^	0.41 ± 0.09 ^d^
**CLF**	10.64 ± 21.07 ^e^	00.00 ± 00.00 ^e^	0.20 ± 0.57 ^f^	165.57 ± 159.40 ^c^	0.42 ± 0.21 ^d^	111.68 ± 22.64 ^a^	105.07 ± 27.30 ^b^	85.99 ± 44.40 ^e^	0.41 ± 0.09 ^d^

Whereas CLL is *C. lancifolius* leaves, CLR is *C. lancifolius* roots, CLF is *C. lancifolius* fruits, CEL is *C. erectus* leaves, CER is *C. erectus* roots, CEF is *C. erectus* fruits. Means with different letters within the same column are significantly different *p* ≤ 0.05.

**Table 4:** Optimum extraction conditions of the most extracted polyphenols from different parts of *Conocarpus* spp.

Component	Solvent Conc. (%)	Temp. (°C)	Time (h)	Plant
Vanillic acid	86.82	57.47	2.13	CER
*p*-Coumaric acid	102.98	36.16	2.27	
Quercetin	82.29	55.06	1.67	
Rutin hydrate	48.46	54.70	2.31	CEL
Flavone	61.46	57.66	1.56	
*t*-Ferulic acid	46.04	71.05	4.05	CLL
Sinapic acid	71.50	59.23	1.95	
Protocatechuic acid	101.37	51.40	2.05	CLF

Whereas CLL is *C. lancifolius* leaves, CLF is *C. lancifolius* fruits, CEL is *C. erectus* leaves, CER is *C. erectus* roots.

## Data Availability

The data presented in this study are available in the Supplementary Materials.

## Supplementary Materials

The following are available online, Figure S1: Flow diagram of extraction process of phytochemicals from *Conocarpus* spp. parts (fruits, leaves and roots), Figure S2: HPLC chromatogram of phytochemicals from *Conocarpus* spp., Table S1: Central composite design arrangement and responses variable of vanillic acid (ppm) at *p* ≤ 0.05, Table S2: Central composite design arrangement and responses variable of *p*-coumaric acid (ppm) at *p* ≤ 0.05, Table S3: Central composite design arrangement and responses variable of *t*-ferulic acid (ppm) at *p* ≤ 0.05, Table S4: Central composite design arrangement and responses variable of sinapic acid (ppm) at *p* ≤ 0.05, Table S5: Central composite design arrangement and responses variable of rutin hydrate (ppm) at *p* ≤ 0.05, Table S6: Central composite design arrangement and responses variable of protocatechuic acid (ppm) at *p* ≤ 0.05, Table S7: Central composite design arrangement and responses variable of quercetin (ppm) at *p* ≤ 0.05, Table S8: Central composite design arrangement and responses variable of flavone (ppm) at *p* ≤ 0.05, Table S9: Botanical classification of *Conocarpus* species, Table S10: Independent variables and their levels used in the response surface design, Table S11: Validation Summary of HPLC, Table S12: LOD and LOQ of polyphenols, Table S13: Retention time (RT) of detected polyphenolic compounds studied in ethanolic extract of *Conocarpus* spp at l = 210 nm.
