# Supplementary material for: Phytochemicals of Conocarpus spp. as a Natural and Safe Source of Phenolic Compounds and Antioxidants
Source: Molecules. 2021 Feb 18;26(4):1069. doi: 10.3390/molecules26041069 (PMC7922956; doi:10.3390/molecules26041069)
Supplement: Supplementary file 1 [file molecules-26-01069-s001.pdf]

Supporting Information

Phytochemicals of *Conocarpus* spp. as a natural and safe source of phenolic compounds and antioxidants

Hanan S. Afifi [1,*], Hassan M. Al Marzooqi [1], Mohammad J. Tabbaa [2,3] and Ahmed A. Arran [2]

[1] Food Research Section, R&D Division, Abu Dhabi Agriculture and Food Safety Authority, P.O. Box 52150, Abu Dhabi, UAE; hassan.marzouqi@adafsa.gov.ae
[2] Agriculture Research Section, R&D Division, Abu Dhabi Agriculture and Food Safety Authority, P.O. Box 52150, Abu Dhabi, UAE; Mjtabbaa@ju.edu.jo (M.J.T.); Ahmedarran@hotmail.com (A.A.A.)
[3] Department of Animal Production, School of Agriculture, The University of Jordan, Amman 11942, Jordan; Mjtabbaa@ju.edu.jo (M.J.T.)
* Correspondence: hanan.afifi@adafsa.gov.ae or hanan.s.afifi@gmail.com; Tel.: +97128181759

Abbreviations

**RSM**	Response Surface Methodology
**CCD**	Central Composite Design
**CL**	*Conocarpus lancifolius*
**CE**	*Conocarpus erectus*
**CLL**	*Conocarpus lancifolius* leaves
**CLF**	*Conocarpus lancifolius* fruits
**CLR**	*Conocarpus lancifolius* roots
**CEL**	*Conocarpus erectus* leaves
**CEF**	*Conocarpus erectus* fruits
**CER**	*Conocarpus erectus* roots

Public

Table S1. Central composite design arrangement and responses variable of vanillic acid (ppm) at $P \leq 0.05$.

Standard Order	Coded variables			*erectus*			*lancifolius*		
	Solvent conc.%	Temp (°C)	Time (h)	Leaves	Roots	Fruits	Leaves	Roots	Fruits
1	1 (100)	1 (65)	0(2)	2.54 ± 0.05	22.87 ± 3.89	0.00 ± 0.00	0.00 ± 0.00	15.62 ± 1.23	1.89 ± 0.11
2	−1 (50)	0 (55)	1(3)	0.00 ± 0.00	10.11 ± 0.20	8.64 ± 0.17	0.00 ± 0.00	20.53 ± 0.72	2.36 ± 0.07
3	0 (75)	0 (55)	0(2)	5.00 ± 0.36	16.40 ± 0.93	7.06 ± 0.53	0.01 ± 0.02	19.97 ± 0.16	0.00 ± 0.00
4	0 (75)	1 (65)	1(3)	2.72 ± 0.11	4.45 ± 0.17	5.15 ± 0.26	0.00 ± 0.00	8.04 ± 0.16	1.23 ± 1.10
5	1(100)	0 (55)	−1(1)	8.63 ± 0.20	1.786 ± 0.14	2.24 ± 0.05	0.00 ± 0.00	1.27 ± 0.02	0.00 ± 0.00
6	1(100)	−1 (45)	0(2)	3.73 ± 0.04	15.40 ± 0.98	4.15 ± 0.19	0.00 ± 0.00	26.40 ± 1.14	0.00 ± 0.00
7	−1(50)	1 (65)	0(2)	8.47 ± 0.21	12.35 ± 0.87	10.78 ± 0.80	0.00 ± 0.00	14.97 ± 0.35	1.46 ± 0.12
8	0 (75)	0 (55)	0(2)	6.22 ± 4.68	18.36 ± 1.00	6.65 ± 0.99	0.00 ± 0.00	15.73 ± 0.63	0.00 ± 0.00
9	0 (75)	1 (65)	−1(1)	0.00 ± 0.00	21.60 ± 1.65	4.27 ± 0.25	0.00 ± 0.00	11.99 ± 2.70	0.00 ± 0.00
10	1(100)	0 (55)	1(3)	0.00 ± 0.00	39.37 ± 0.76	12.69 ± 0.27	4.18 ± 0.32	20.26 ± 1.04	0.00 ± 0.00
11	0 (75)	−1 (45)	1(3)	5.05 ± 0.06	29.21 ± 0.71	3.50 ± 0.25	3.04 ± 0.27	15.12 ± 0.39	0.00 ± 0.00
12	0 (75)	0 (55)	0(2)	4.00 ± 0.11	16.06 ± 0.30	9.74 ± 0.24	0.00 ± 0.00	15.41 ± 0.57	0.00 ± 0.00
13	−1(50)	0 (55)	−1(1)	3.73 ± 0.40	0.00 ± 0.00	2.02 ± 0.03	2.09 ± 0.09	36.57 ± 1.31	0.00 ± 0.00
14	0 (75)	−1 (45)	−1(1)	0.00 ± 0.00	8.96 ± 0.25	7.57 ± 0.39	0.00 ± 0.00	0.00 ± 0.00	0.00 ± 0.00
15	−1(50)	−1 (45)	0(2)	3.94 ± 0.19	**124.79 ± 3.80**	23.17 ± 0.21	7.22 ± 0.47	**44.48 ±** 2.04	0.00 ± 0.00

Values are expressed as mean ± standard deviation (n = 3).

Public

Table S2. Central composite design arrangement and responses variable of *p*-coumaric acid (ppm) at $P \leq 0.05$.

Standard Order	Coded variables			*erectus*			*lancifolius*		
	Solvent conc.%	Temp (°C)	Time (h)	Leaves	Roots	Fruits	Leaves	Roots	Fruits
1	1 (100)	1 (65)	0(2)	0.02 ± 0.02	3.58 ± 1.24	37.49 ± 4.31	5.36 ± 0.41	2.82 ± 0.12	38.41 ± 1.69
2	−1 (50)	0 (55)	1(3)	4.96 ± 0.06	0.78 ± 0.04	0.00 ± 0.00	74.48 ± 2.87	2.59 ± 0.02	0.00 ± 0.00
3	0 (75)	0 (55)	0(2)	14.74 ± 0.07	0.00 ± 0.00	0.00 ± 0.00	16.68 ± 1.91	4.16 ± 0.14	15.65 ± 0.33
4	0 (75)	1 (65)	1(3)	6.80 ± 2.24	0.00 ± 0.00	0.00 ± 0.00	45.48 ± 11.79	14.97 ± 0.91	27.00 ± 6.30
5	1(100)	0 (55)	−1(1)	115.46 ± 2.36	1.76 ± 0.06	9.98 ± 0.87	20.37 ± 1.56	3.00 ± 0.10	21.57 ± 1.75
6	1(100)	−1 (45)	0(2)	0.00 ± 0.00	0.84 ± 0.04	0.00 ± 0.00	1.80 ± 0.69	4.16 ± 0.14	4.88 ± 0.13
7	−1(50)	1 (65)	0(2)	3.40 ± 0.01	1.13 ± 0.03	1.11 ± 0.13	44.22 ± 6.74	3.34 ± 0.12	1.03 ± 0.04
8	0 (75)	0 (55)	0(2)	19.90 ± 5.13	0.00 ± 0.00	0.00 ± 0.00	18.81 ± 2.23	3.79 ± 0.13	18.72 ± 0.50
9	0 (75)	1 (65)	−1(1)	10.17 ± 0.09	8.97 ± 0.07	0.63 ± 0.05	1.06 ± 0.05	1.20 ± 0.82	0.00 ± 0.00
10	1(100)	0 (55)	1(3)	**134.14 ± 1.99**	13.61 ± 0.54	42.47 ± 2.32	0.21 ± 0.02	19.79 ± 0.64	7.76 ± 0.25
11	0 (75)	−1 (45)	1(3)	12.22 ± 0.74	4.57 ± 0.05	1.37 ± 0.14	2.01 ± 1.81	1.47 ± 0.04	2.56 ± 0.05
12	0 (75)	0 (55)	0(2)	18.46 ± 0.56	0.00 ± 0.00	0.00 ± 0.00	15.05 ± 0.21	3.08 ± 0.09	15.47 ± 1.13
13	−1(50)	0 (55)	−1(1)	1.63 ± 0.05	2.25 ± 0.06	0.63 ± 0.03	3.42 ± 1.26	4.93 ± 0.21	10.34 ± 0.79
14	0 (75)	−1 (45)	−1(1)	1.96 ± 0.36	0.47 ± 0.03	1.90 ± 0.28	0.00 ± 0.00	0.00 ± 0.00	1.01 ± 0.02
15	−1(50)	−1 (45)	0(2)	1.95 ± 0.41	**235.06 ± 5.00**	0.00 ± 0.00	2.59 ± 0.40	46.38 ± 1.40	0.00 ± 0.00

Values are expressed as mean ± standard deviation ($n = 3$).

Public

Table S3. Central composite design arrangement and responses variable of *t*-ferulic acid (ppm) at $P \leq 0.05$.

Standard Order	Coded variables			*erectus*			*lancifolius*		
	Solvent conc.%	Temp (°C)	Time (h)	Leaves	Roots	Fruits	Leaves	Roots	Fruits
1	1 (100)	1 (65)	0(2)	2.67 ± 0.04	4.14 ± 1.42	0.16 ± 0.15	0.00 ± 0.00	2.55 ± 0.09	0.00 ± 0.00
2	−1 (50)	0 (55)	1(3)	51.80 ± 1.53	0.41 ± 0.01	1.79 ± 0.37	0.00 ± 0.00	3.72 ± 0.18	0.00 ± 0.00
3	0 (75)	0 (55)	0(2)	27.03 ± 0.25	1.22 ± 0.02	1.03 ± 0.06	0.00 ± 0.00	1.40 ± 0.03	0.00 ± 0.00
4	0 (75)	1 (65)	1(3)	19.19 ± 0.17	0.00 ± 0.00	0.00 ± 0.00	0.00 ± 0.00	0.00 ± 0.00	0.14 ± 0.13
5	1(100)	0 (55)	−1(1)	31.86 ± 1.07	1.11 ± 0.02	0.00 ± 0.00	3.96 ± 0.10	0.00 ± 0.00	0.00 ± 0.00
6	1(100)	−1 (45)	0(2)	107.45 ± 1.97	0.96 ± 0.14	4.21 ± 0.56	122.75 ± 5.73	6.36 ± 0.06	0.00 ± 0.00
7	−1(50)	1 (65)	0(2)	31.40 ± 0.37	1.20 ± 0.02	1.02 ± 0.03	0.00 ± 0.00	0.80 ± 0.03	1.56 ± 0.08
8	0 (75)	0 (55)	0(2)	23.44 ± 10.50	1.37 ± 0.41	1.38 ± 0.30	0.00 ± 0.00	2.02 ± 0.02	0.00 ± 0.00
9	0 (75)	1 (65)	−1(1)	54.44 ± 0.82	1.71 ± 0.06	1.20 ± 0.06	1.09 ± 0.11	0.46 ± 0.04	0.00 ± 0.00
10	1(100)	0 (55)	1(3)	45.33 ± 1.81	1.83 ± 0.08	2.28 ± 0.16	**175.20 ± 1.72**	8.59 ± 0.35	0.00 ± 0.00
11	0 (75)	−1 (45)	1(3)	37.25 ± 3.03	3.03 ± 0.06	0.00 ± 0.00	0.35 ± 0.31	1.08 ± 0.07	1.55 ± 0.10
12	0 (75)	0 (55)	0(2)	27.13 ± 0.27	1.41 ± 0.05	1.00 ± 0.13	0.00 ± 0.00	1.51 ± 0.17	0.00 ± 0.00
13	−1(50)	0 (55)	−1(1)	0.84 ± 0.04	0.61 ± 0.02	0.83 ± 0.05	32.01 ± 1.07	1.50 ± 0.17	0.00 ± 0.00
14	0 (75)	−1 (45)	−1(1)	0.00 ± 0.00	0.00 ± 0.00	1.55 ± 0.32	22.42 ± 2.44	0.00 ± 0.00	1.63 ± 0.09
15	−1(50)	−1 (45)	0(2)	1.34 ± 0.08	**137.07 ± 1.29**	4.10 ± 0.10	37.74 ± 1.07	50.16 ± 1.23	0.00 ± 0.00

Values are expressed as mean ± standard deviation ($n = 3$).

Public

Table S4. Central composite design arrangement and responses variable of sinapic acid (ppm) at $P \leq 0.05$.

Standard Order	Coded variables			*erectus*			*lancifolius*		
	Solvent conc.%	Temp (°C)	Time (h)	Leaves	Roots	Fruits	Leaves	Roots	Fruits
1	1 (100)	1 (65)	0(2)	0.00 ± 0.00	49.98 ± 24.44	0.00 ± 0.00	29.76 ± 0.39	0.00 ± 0.00	24.10 ± 1.99
2	−1 (50)	0 (55)	1(3)	94.32 ± 0.82	24.14 ± 0.17	15.38 ± 1.51	21.35 ± 1.56	11.20 ± 0.74	0.00 ± 0.00
3	0 (75)	0 (55)	0(2)	67.27 ± 1.01	0.00 ± 0.00	31.00 ± 0.46	42.46 ± 3.37	0.00 ± 0.00	0.00 ± 0.00
4	0 (75)	1 (65)	1(3)	49.48 ± 0.32	32.33 ± 2.46	0.00 ± 0.00	68.97 ± 14.60	0.00 ± 0.00	0.31 ± 0.29
5	1(100)	0 (55)	−1(1)	93.02 ± 1.01	5.28 ± 0.04	0.00 ± 0.00	115.79 ± 6.01	0.00 ± 0.00	0.00 ± 0.00
6	1(100)	−1 (45)	0(2)	0.00 ± 0.00	31.78 ± 1.94	0.00 ± 0.00	**254.54 ± 38.75**	29.94 ± 0.29	0.00 ± 0.00
7	−1(50)	1 (65)	0(2)	63.45 ± 0.45	0.00 ± 0.00	21.13 ± 1.24	49.99 ± 8.31	28.95 ± 0.37	0.00 ± 0.00
8	0 (75)	0 (55)	0(2)	66.78 ± 4.33	0.00 ± 0.00	27.36 ± 0.49	45.56 ± 2.91	0.00 ± 0.00	0.00 ± 0.00
9	0 (75)	1 (65)	−1(1)	81.89 ± 1.66	0.00 ± 0.00	17.29 ± 0.60	21.24 ± 2.31	4.67 ± 2.11	0.00 ± 0.00
10	1(100)	0 (55)	1(3)	189.20 ± 3.14	0.00 ± 0.00	53.31 ± 2.95	17.09 ± 0.17	0.00 ± 0.00	0.00 ± 0.00
11	0 (75)	−1 (45)	1(3)	45.56 ± 3.04	0.00 ± 0.00	0.00 ± 0.00	37.01 ± 4.90	0.00 ± 0.00	76.91 ± 3.01
12	0 (75)	0 (55)	0(2)	63.17 ± 1.27	0.00 ± 0.00	35.22 ± 1.33	41.69 ± 1.19	0.00 ± 0.00	0.00 ± 0.00
13	−1(50)	0 (55)	−1(1)	18.25 ± 0.80	0.00 ± 0.00	7.35 ± 0.67	47.20 ± 4.35	61.93 ± 1.61	0.00 ± 0.00
14	0 (75)	−1 (45)	−1(1)	17.22 ± 0.66	0.00 ± 0.00	21.13 ± 1.88	40.43 ± 1.55	0.00 ± 0.00	28.02 ± 2.44
15	−1(50)	−1 (45)	0(2)	16.41 ± 1.23	**237.54 ± 3.58**	58.84 ± 3.67	41.89 ± 2.82	39.83 ± 0.63	0.00 ± 0.00

Values are expressed as mean ± standard deviation ($n = 3$).

Public

Table S5. Central composite design arrangement and responses variable of rutin hydrate (ppm) at $P \leq 0.05$.

Standard Order	Coded variables			*erectus*			*lancifolius*		
	Solvent conc.%	Temp (°C)	Time (h)	Leaves	Roots	Fruits	Leaves	Roots	Fruits
1	1 (100)	1 (65)	0(2)	133.34 ± 1.35	157.21 ± 37.50	5.09 ± 2.71	554.89 ± 13.69	29.34 ± 0.86	35.54 ± 1.71
2	−1 (50)	0 (55)	1(3)	65.40 ± 0.57	5.98 ± 0.23	8.18 ± 0.19	18.10 ± 2.01	13.77 ± 0.82	139.12 ± 3.24
3	0 (75)	0 (55)	0(2)	127.48 ± 0.47	226.07 ± 5.68	10.00 ± 0.00	185.98 ± 5.86	10.18 ± 0.24	267.93 ± 2.45
4	0 (75)	1 (65)	1(3)	38.11 ± 0.11	11.09 ± 0.89	13.88 ± 0.23	228.90 ± 27.26	217.82 ± 2.56	31.50 ± 4.44
5	1(100)	0 (55)	−1(1)	413.41 ± 2.20	25.30 ± 1.18	10.68 ± 0.76	272.14 ± 13.80	59.14 ± 1.18	84.37 ± 3.80
6	1(100)	−1 (45)	0(2)	167.43 ± 3.09	47.74 ± 1.23	8.69 ± 0.43	475.12 ± 83.75	80.58 ± 1.29	68.22 ± 1.12
7	−1(50)	1 (65)	0(2)	62.54 ± 0.47	6.84 ± 0.06	9.87 ± 0.41	78.38 ± 10.23	115.17 ± 1.82	104.22 ± 1.29
8	0 (75)	0 (55)	0(2)	111.47 ± 5.60	220.66 ± 2.35	9.14 ± 0.28	192.30 ± 4.50	10.32 ± 0.57	275.69 ± 5.82
9	0 (75)	1 (65)	−1(1)	250.27 ± 2.23	399.30 ± 2.36	14.31 ± 0.74	29.08 ± 1.55	3.76 ± 1.02	222.64 ± 7.23
10	1(100)	0 (55)	1(3)	**1362.55 ± 8.12**	29.67 ± 0.85	27.37 ± 3.46	102.40 ± 2.81	19.53 ± 0.72	458.59 ± 3.44
11	0 (75)	−1 (45)	1(3)	223.19 ± 4.37	136.47 ± 2.68	5.67 ± 0.38	94.01 ± 3.96	15.09 ± 0.08	30.17 ± 0.99
12	0 (75)	0 (55)	0(2)	131.34 ± 4.39	249.80 ± 1.01	11.44 ± 1.27	179.70 ± 1.64	9.15 ± 4.77	258.21 ± 5.54
13	−1(50)	0 (55)	−1(1)	23.90 ± 1.67	0.00 ± 0.00	7.37 ± 0.28	91.43 ± 2.36	85.26 ± 3.63	17.19 ± 1.80
14	0 (75)	−1 (45)	−1(1)	33.99 ± 4.00	21.48 ± 0.51	19.58 ± 1.46	59.86 ± 3.14	6.36 ± 0.40	140.18 ± 3.74
15	−1(50)	−1 (45)	0(2)	30.45 ± 2.15	635.60 ± 112.26	19.59 ± 0.69	99.35 ± 8.41	85.61 ± 0.63	40.08 ± 1.21

Values are expressed as mean ± standard deviation ($n = 3$).

Public

Table S6. Central composite design arrangement and responses variable of protocatechuic acid (ppm) at $P \leq 0.05$.

Standard Order	Coded variables			*erectus*			*lancifolius*		
	Solvent conc.%	Temp (°C)	Time (h)	Leaves	Roots	Fruits	Leaves	Roots	Fruits
1	1 (100)	1 (65)	0(2)	108.55 ± 0.85	39.72 ± 2.88	80.71 ± 3.94	21.15 ± 2.86	12.25 ± 1.11	99.23 ± 3.35
2	−1 (50)	0 (55)	1(3)	99.48 ± 0.85	32.74 ± 1.56	95.10 ± 2.66	25.18 ± 1.81	42.25 ± 1.92	99.59 ± 0.72
3	0 (75)	0 (55)	0(2)	13.38 ± 0.26	89.67 ± 1.54	116.77 ± 3.82	23.44 ± 2.49	39.67 ± 1.00	116.14 ± 2.40
4	0 (75)	1 (65)	1(3)	9.59 ± 0.05	7.32 ± 0.48	3.47 ± 0.11	42.77 ± 2.51	2.61 ± 0.24	127.39 ± 14.86
5	1(100)	0 (55)	−1(1)	107.11 ± 1.13	16.28 ± 0.54	100.79 ± 1.09	36.59 ± 2.57	5.11 ± 0.12	107.24 ± 3.46
6	1(100)	−1 (45)	0(2)	7.85 ± 0.17	32.94 ± 2.05	105.08 ± 3.66	119.11 ± 19.63	8.84 ± 0.25	99.40 ± 0.97
7	−1(50)	1 (65)	0(2)	14.89 ± 0.03	35.39 ± 2.02	101.41 ± 2.41	19.56 ± 2.40	10.20 ± 0.26	132.16 ± 6.31
8	0 (75)	0 (55)	0(2)	18.91 ± 1.58	76.34 ± 0.60	125.40 ± 26.67	21.54 ± 1.47	34.76 ± 0.60	110.54 ± 1.63
9	0 (75)	1 (65)	−1(1)	23.63 ± 0.25	35.74 ± 1.13	72.85 ± 49.84	21.57 ± 1.00	38.71 ± 4.50	98.98 ± 8.66
10	1(100)	0 (55)	1(3)	20.40 ± 1.73	71.54 ± 1.73	105.13 ± 1.80	18.67 ± 1.10	28.52 ± 0.73	109.74 ± 2.39
11	0 (75)	−1 (45)	1(3)	12.15 ± 0.13	99.51 ± 0.86	106.79 ± 6.16	13.52 ± 4.02	8.35 ± 0.37	104.51 ± 2.13
12	0 (75)	0 (55)	0(2)	15.28 ± 0.40	79.63 ± 0.81	70.47 ± 49.61	23.95 ± 1.71	37.77 ± 0.51	108.79 ± 1.57
13	−1(50)	0 (55)	−1(1)	17.69 ± 1.12	41.27 ± 1.43	107.87 ± 2.66	15.05 ± 1.18	40.77 ± 1.34	90.73 ± 1.70
14	0 (75)	−1 (45)	−1(1)	8.11 ± 0.58	7.92 ± 0.07	130.79 ± 2.23	20.71 ± 1.26	42.13 ± 1.78	101.50 ± 1.57
15	−1(50)	−1 (45)	0(2)	12.29 ± 1.02	53.02 ± 2.25	101.41 ± 1.83	14.70 ± 0.98	102.21 ± 2.03	**183.40 ± 4.33**

Values are expressed as mean ± standard deviation ($n = 3$).

Public

Table S7. Central composite design arrangement and responses variable of quercetin (ppm) at $P \leq 0.05$.

Standard Order	Coded variables			*erectus*			*lancifolius*		
	Solvent conc.%	Temp (°C)	Time (h)	Leaves	Roots	Fruits	Leaves	Roots	Fruits
1	1 (100)	1 (65)	0(2)	5.49 ± 0.34	111.71 ± 1.59	112.23 ± 2.52	5.99 ± 0.30	4.59 ± 0.25	117.67 ± 3.20
2	−1 (50)	0 (55)	1(3)	111.97 ± 0.98	111.11 ± 1.86	120.77 ± 2.01	6.59 ± 0.42	110.75 ± 1.42	116.15 ± 2.55
3	0 (75)	0 (55)	0(2)	5.20 ± 0.36	104.91 ± 1.18	116.38 ± 4.67	6.77 ± 0.47	114.49 ± 1.25	104.68 ± 7.85
4	0 (75)	1 (65)	1(3)	5.36 ± 0.06	116.35 ± 2.10	119.71 ± 0.85	5.21 ± 1.82	121.41 ± 1.42	117.98 ± 3.86
5	1(100)	0 (55)	−1(1)	8.17 ± 0.11	112.75 ± 2.80	117.26 ± 0.80	5.93 ± 0.21	4.74 ± 0.35	82.23 ± 1.93
6	1(100)	−1 (45)	0(2)	8.59 ± 0.55	115.35 ± 0.56	122.23 ± 1.28	19.87 ± 2.79	107.49 ± 2.76	10.48 ± 1.56
7	−1(50)	1 (65)	0(2)	6.18 ± 0.16	115.17 ± 2.48	122.53 ± 5.67	5.91 ± 0.80	0.00 ± 0.00	119.23 ± 2.14
8	0 (75)	0 (55)	0(2)	8.57 ± 2.01	100.12 ± 0.83	112.95 ± 1.35	6.96 ± 0.45	117.41 ± 1.35	108.54 ± 3.64
9	0 (75)	1 (65)	−1(1)	5.62 ± 0.33	106.77 ± 1.10	110.64 ± 2.04	5.81 ± 0.86	112.19 ± 9.36	120.92 ± 3.86
10	1(100)	0 (55)	1(3)	5.82 ± 0.76	87.73 ± 1.18	4.02 ± 0.11	109.51 ± 1.84	109.68 ± 2.53	110.46 ± 2.19
11	0 (75)	−1 (45)	1(3)	4.60 ± 0.07	102.16 ± 0.99	102.72 ± 2.50	7.26 ± 2.32	108.23 ± 1.73	116.23 ± 3.17
12	0 (75)	0 (55)	0(2)	5.45 ± 1.27	108.92 ± 1.08	115.18 ± 3.76	5.40 ± 0.41	113.88 ± 1.06	110.42 ± 4.61
13	−1(50)	0 (55)	−1(1)	5.08 ± 0.30	113.58 ± 2.48	115.16 ± 1.99	3.93 ± 0.12	114.03 ± 0.51	110.19 ± 2.07
14	0 (75)	−1 (45)	−1(1)	4.41 ± 0.45	101.22 ± 1.19	120.88 ± 1.82	5.19 ± 0.36	5.54 ± 0.31	113.56 ± 1.76
15	−1(50)	−1 (45)	0(2)	5.04 ± 0.09	**137.31 ± 3.58**	115.86 ± 4.69	6.65 ± 0.61	108.95 ± 2.25	117.32 ± 2.58

Values are expressed as mean ± standard deviation ($n = 3$).

Public

Table S8. Central composite design arrangement and responses variable of flavone (ppm) at $P \leq 0.05$.

Standard Order	Coded variables			*erectus*			*lancifolius*		
	Solvent conc.%	Temp (°C)	Time (h)	Leaves	Roots	Fruits	Leaves	Roots	Fruits
1	1 (100)	1 (65)	0(2)	122.27 ± 1.97	115.67 ± 10.445	115.71 ± 10.30	78.94 ± 6.10	85.15 ± 1.13	101.02 ± 2.45
2	−1 (50)	0 (55)	1(3)	338.08 ± 1.82	118.42 ± 1.380	100.71 ± 2.93	0.00 ± 0.00	77.28 ± 1.66	101.87 ± 1.89
3	0 (75)	0 (55)	0(2)	532.00 ± 0.14	152.13 ± 1.305	103.86 ± 1.83	367.06 ± 2.05	99.10 ± 1.57	70.63 ± 5.28
4	0 (75)	1 (65)	1(3)	706.39 ± 5.79	169.70 ± 1.572	100.34 ± 11.23	123.81 ± 11.41	70.49 ± 0.71	98.38 ± 16.87
5	1(100)	0 (55)	−1(1)	**2119.46 ± 4.12**	249.97 ± 7.556	125.59 ± 4.62	66.70 ± 11.50	72.92 ± 3.69	107.78 ± 2.03
6	1(100)	−1 (45)	0(2)	502.41 ± 3.08	111.30 ± 2.945	91.22 ± 2.40	0.00 ± 0.00	81.65 ± 1.99	0.00 ± 0.00
7	−1(50)	1 (65)	0(2)	948.88 ± 2.43	138.85 ± 1.232	124.26 ± 8.14	123.88 ± 6.18	0.00 ± 0.00	0.00 ± 0.00
8	0 (75)	0 (55)	0(2)	541.31 ± 152.17	163.06 ± 3.874	92.15 ± 13.02	125.26 ± 9.54	96.35 ± 1.20	82.15 ± 1.82
9	0 (75)	1 (65)	−1(1)	177.87 ± 2.41	105.58 ± 2.000	138.58 ± 2.30	122.34 ± 4.51	96.32 ± 19.03	72.64 ± 1.93
10	1(100)	0 (55)	1(3)	1813.21 ± 8.14	266.13 ± 5.369	140.18 ± 2.33	413.48 ± 12.36	89.64 ± 1.15	184.88 ± 7.06
11	0 (75)	−1 (45)	1(3)	150.85 ± 2.20	89.08 ± 1.602	131.17 ± 2.69	120.11 ± 20.64	75.70 ± 1.43	79.80 ± 1.67
12	0 (75)	0 (55)	0(2)	560.11 ± 21.90	159.06 ± 0.493	96.77 ± 5.01	376.11 ± 10.70	100.43 ± 1.41	79.96 ± 0.58
13	−1(50)	0 (55)	−1(1)	124.82 ± 13.69	68.66 ± 1.464	97.86 ± 2.40	569.61 ± 16.22	102.41 ± 2.12	75.45 ± 2.23
14	0 (75)	−1 (45)	−1(1)	62.28 ± 4.31	77.48 ± 2.665	93.20 ± 2.68	744.68 ± 30.81	90.90 ± 2.70	71.13 ± 2.36
15	−1(50)	−1 (45)	0(2)	87.11 ± 7.06	121.81 ± 2.112	93.18 ± 2.87	92.12 ± 4.51	81.69 ± 1.98	103.11 ± 1.02

Values are expressed as mean ± standard deviation ($n = 3$).

Public

Table S9. Botanical classification of *Conocarpus* species

**Species (1)**	**Species (2)**
Kingdom: Plantae	Kingdom: Plantae
Clade: Angiosperms	Clade: Angiosperms
Clade: Eudicots	Clade: Eudicots
Clade: Eudicots	Clade: Rosids
Order: Myrtales	Order: Myrtales
Family: Combretaceae	Family: Combretaceae
Genus: Conocarpus L.	Genus: Conocarpus L.
Species: *C. lancifolius*	Species: *C. erectus*
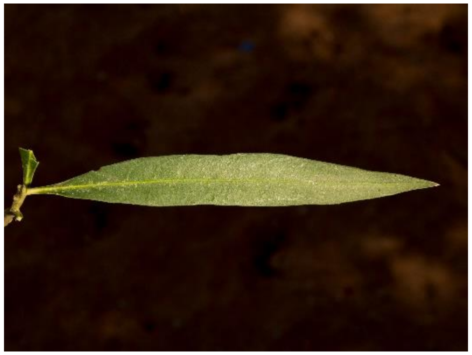	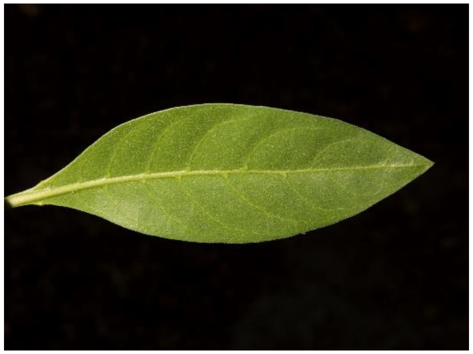
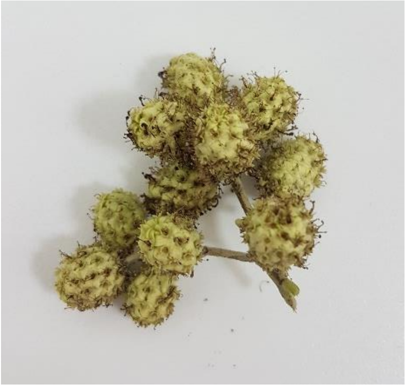	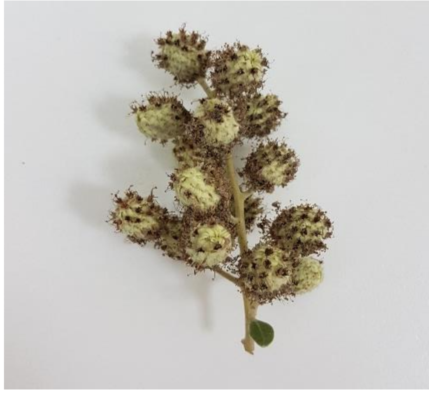
**CL**	**CE**

Public

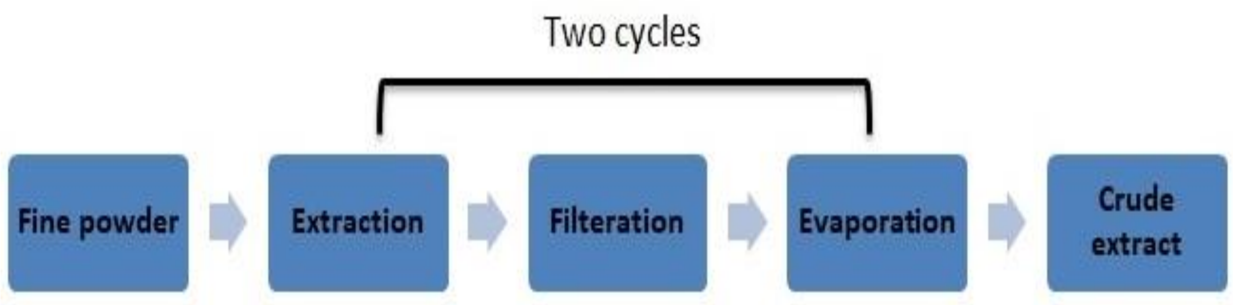

Figure S1. Flow diagram of extraction process of phytochemicals from *Conocarpus* spp. parts (fruits, leaves and roots).

Table S10. Independent variables and their levels used in the response surface design.

Independent variables	Unit	Symbol	Code Levels ($\mathcal{X}$i)		
			**−1**	**0**	**1**
**Concentration of solvent**	% (*v*/*v*)	X_1	50	75	100
**Temperature**	°C	X_2	45	55	65
**Time**	h	X_3	1	2	3

Public

Table S11. Validation Summary of HPLC

Parameter	Acceptance Criteria	Results
Specificity	≤ 30% of LOQ	No Peak Observed
Selectivity& system reliability	RSD < 10%	RSD < 10%
Linearity	$R^2 > 0.9900$	$R^2 > 0.9961–0.9983$
Matrix effect	< 20%	< 20%
LOQ	-	0.04–1.16 μg/mL
LOD	-	0.01–0.02 μg/mL
Recovery	70–120%	70–120%
Repeatability	% Recovery:70–120%<br>% RSD: < 20%	% Recovery:70–120%<br>% RSD: < 20%
Range	-	10–200 μg/mL

Table S12. LOD and LOQ of polyphenols

**Compounds**	**LOD (μg/mL)**	**LOQ (μg/mL)**
2-Hexenal	0.024	1.160
a-Pinene	0.023	0.99
Camphene	0.022	0.085
4-hydroxy benzoic acid	0.011	0.071
Vanillic acid	0.019	0.042
Caffeic acid	0.010	0.062
Salicylic acid	0.016	0.035
1,2-dihydroxy benzene	0.012	0.063
Catechin	0.013	0.042
Benzoic acid	0.011	0.060
p-Coumaric acid	0.010	0.053
t-Ferulic acid	0.010	0.064
Sinapic acid	0.016	0.067
Vanillin	0.015	0.050
Chlorogenic acid	0.010	0.074
Rutin hydrate	0.010	0.052
Cinnamic acid	0.010	0.140
t-Cinnamic acid	0.010	0.110
Protocatechuic acid	0.011	0.029
Quercetin	0.011	0.047
Flavone	0.012	0.055

Public

Table S13.
Retention time (RT) of detected polyphenolic compounds studied in ethanolic extract of *Conocarpus* spp at λ =210 nm.

No	Compounds	RT (min)
1.	2-Hexenal	2.98
2.	*a*-Pinene	3.35
3.	Camphene	3.79
4.	4-hydroxy benzoic acid	3.90, 3.95, 4.24
5.	Vanillic acid	4.59, 4.71
6.	Caffeic acid	4.82, 4.87,4.99
7.	Salicylic acid	5.44, 5.81
8.	1,2-dihydroxy benzene	6.30, 6.49, 6.57
9.	Catechin	6.85, 6.96, 7.05
10.	Benzoic acid	7.16, 7.38, 7.43, 7.47
11.	*p*-Coumaric acid	7.74, 7.76, 7.78
12.	*t*-Ferulic acid	7.84, 7.98, 8.02, 8.29
13.	Sinapic acid	8.35, 8.52, 8.53
14.	Vanillin	8.76
15.	Cinnamic acid	8.82, 8.90, 8.96
16.	Chlorogenic acid	8.60, 8.62
17.	Rutin hydrate	9.22, 9.43, 9.70, 9.72, 10.38
18.	*t*-Cinnamic acid	10.70, 10.96, 11.07, 11.22
19.	Protocatechuic acid	11.51, 11.60, 11.68, 11.70
20.	Quercetin	11.99, 12.11, 12.17, 12.29
21.	Flavone	12.82, 13.02, 13.05

Public

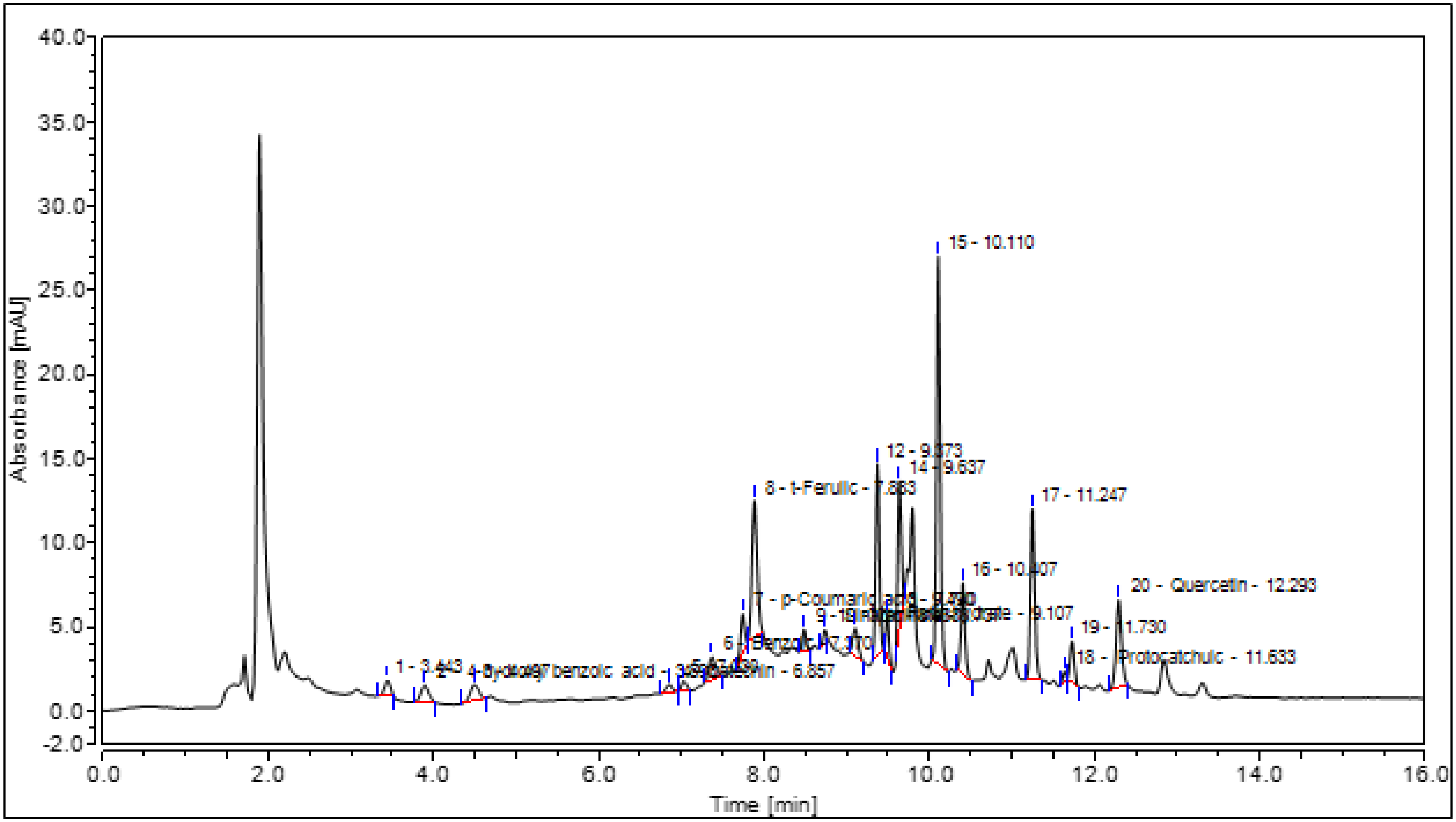

Figure S2. HPLC chromatogram of phytochemicals from *Conocarpus* spp.
